# Overexpressed TP73 induces apoptosis in medulloblastoma

**DOI:** 10.1186/1471-2407-7-127

**Published:** 2007-07-12

**Authors:** Robert C Castellino, Massimiliano De Bortoli, Linda L Lin, Darlene G Skapura, Jessen A Rajan, Adekunle M Adesina, Laszlo Perlaky, Meredith S Irwin, John YH Kim

**Affiliations:** 1 Texas Children's Cancer Center, Department of Pediatrics, Baylor College of Medicine, Houston, TX, 77030, USA; 2 Department of Pathology, Baylor College of Medicine, Houston, TX, 77030, USA; 3 Hospital for Sick Children, Department of Pediatrics, University of Toronto, Toronto, ON, M5G 1X8, Canada

## Abstract

**Background:**

Medulloblastoma is the most common malignant brain tumor of childhood. Children who relapse usually die of their disease, which reflects resistance to radiation and/or chemotherapy. Improvements in outcome require a better understanding of the molecular basis of medulloblastoma growth and treatment response. *TP73 *is a member of the *TP53 *tumor suppressor gene family that has been found to be overexpressed in a variety of tumors and mediates apoptotic responses to genotoxic stress. In this study, we assessed expression of *TP73 *RNA species in patient tumor specimens and in medulloblastoma cell lines, and manipulated expression of full-length TAp73 and amino-terminal truncated ΔNp73 to assess their effects on growth.

**Methods:**

We analyzed medulloblastoma samples from thirty-four pediatric patients and the established medulloblastoma cell lines, Daoy and D283MED, for expression of *TP73 *RNA including the full-length transcript and the 5'-terminal variants that encode the ΔNp73 isoform, as well as *TP53 *RNA using quantitative real time-RTPCR. Protein expression of TAp73 and ΔNp73 was quantitated with immunoblotting methods. Clinical outcome was analyzed based on *TP73 *RNA and p53 protein expression. To determine effects of overexpression or knock-down of TAp73 and ΔNp73 on cell cycle and apoptosis, we analyzed transiently transfected medulloblastoma cell lines with flow cytometric and TUNEL methods.

**Results:**

Patient medulloblastoma samples and cell lines expressed full-length and 5'-terminal variant *TP73 *RNA species in 100-fold excess compared to non-neoplastic brain controls. Western immunoblot analysis confirmed their elevated levels of TAp73 and amino-terminal truncated ΔNp73 proteins. Kaplan-Meier analysis revealed trends toward favorable overall and progression-free survival of patients whose tumors display TAp73 RNA overexpression. Overexpression of TAp73 or ΔNp73 induced apoptosis under basal growth conditions *in vitro *and sensitized them to cell death in response to chemotherapeutic agents.

**Conclusion:**

These results indicate that primary medulloblastomas express significant levels of *TP73 *isoforms, and suggest that they can modulate the survival and genotoxic responsiveness of medulloblastomas cells.

## Background

Medulloblastoma is the most common malignant brain tumor of childhood [1,2]. Treatment with surgery, radiation, and chemotherapy successfully cures many patients, but survivors can suffer significant long-term toxicities affecting their neurocognitive and growth potential [3]. Despite clinical advances, up to 30% of children with medulloblastoma experience tumor progression or recurrence, for which no curative therapy exists. The lack of more effective, less toxic therapies stems from our imperfect understanding of the molecular processes that underlie medulloblastoma growth.

Although the *TP53 *tumor suppressor gene (17p13.1) is mutated in approximately half of human malignancies, it is rarely mutated in medulloblastoma [4-7]. However, several lines of evidence suggest that the *TP53 *pathway is perturbed in medulloblastoma. Frank *et al*. have described abnormalities of the p53-p14^ARF ^pathway in the large cell/anaplastic variant of medulloblastoma [8,9]. Our collaborators have noted significant nuclear p53 immunopositivity consistent with its accumulation and potential mutation in approximately 50% of primary human medulloblastoma examined [A. Adesina, personal communication]. Deletion of the murine homolog, *Trp53*, in the *Patched *haploinsufficient (*Ptch+/-*) mouse model increases the incidence of spontaneous medulloblastoma from approximately 15% to 100% [10]. Individuals with Li-Fraumeni syndrome who carry germline *TP53 *mutations are at increased risk for developing medulloblastoma, but fewer than 10% of sporadic medulloblastoma display *TP53 *mutations [11,12]. There is also evidence that the activity of p53 is regulated by alternate mechanisms as observed in other cancers with *wild-type TP53 *[4].

**Figure 1:**
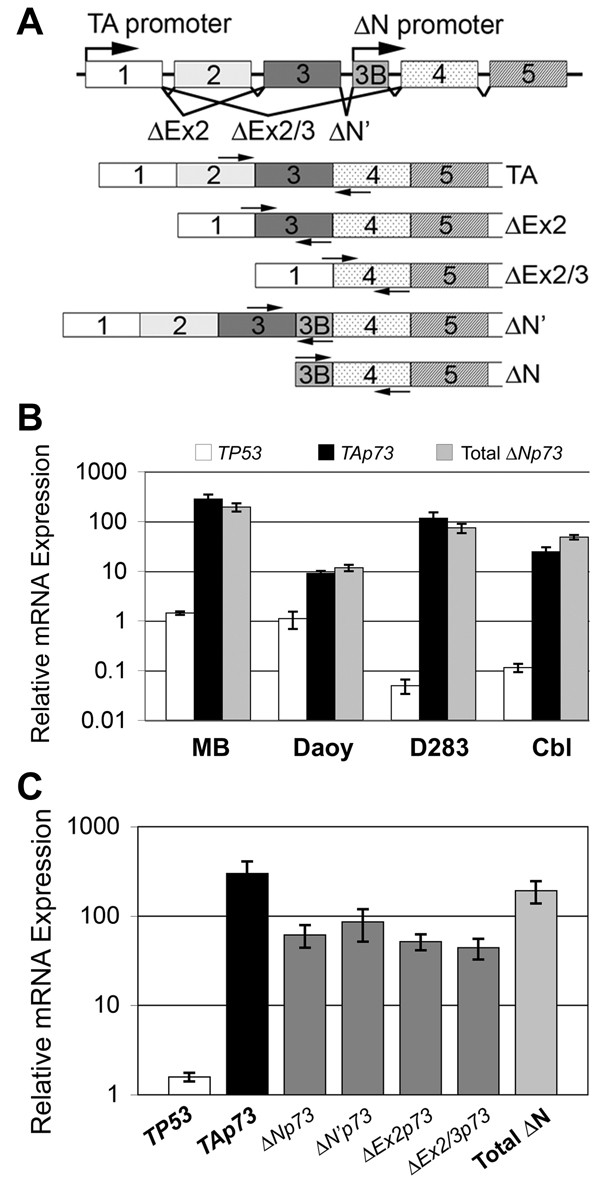
**Primary medulloblastoma specimens and medulloblastoma cell lines overexpress TAp73 and ΔNp73 RNA species relative to *TP53***. **(A) ***TP73 *gene and relative location of isoform-specific primers for qRT-RTPCR of *TP73*. Exons are depicted as boxes with overlying arrows corresponding to location of primers for PCR. **(B) **Established medulloblastoma cell lines, Daoy and D283MED (D283), and human adult cerebellum (Cbl) express high levels of TAp73 and amino-terminal splice variants encoding ΔNp73 in comparison to human fetal brain. Primary medulloblastoma (MB) samples from patients (n = 34) display similar overexpression of TAp73 and ΔNp73 RNA species. **(C) **Primary medulloblastoma samples from patients (n = 34) display overexpression of TAp73 and amino-terminal truncated *TP73 *RNA variants, relative to human fetal brain and normalized to *GAPDH *expression. By comparison, *TP53 *RNA is relatively underexpressed. Total ΔNp73 represents the sum of expression of all amino-terminal-truncated *TP73 *RNA variants (ΔNp73, ΔN'p73, ΔEx2p73, and ΔEx2/3p73). ***Columns***, mean expression of at least 2 experiments; ***error bars***, ± S.E.M. ***Y-axis***, RNA expression relative to human fetal brain and normalized to *GAPDH *expression (N.B. log-scale).

While mutation of *TP53 *itself might not be frequently involved, genes related to *TP53 *may play a role in medulloblastoma [8]. *TP73 *is a member of the *TP53 *gene family. Full-length *TP73*-encoded protein, TAp73, is similar in structure and shares functions with p53 as a candidate tumor suppressor. It is able to activate p53-responsive genes and to induce apoptosis *in vitro *[13]. Differential transcription and alternative splicing of *TP73 *gives rise to at least 35 distinct RNA species and 14 described protein isoforms. Several 5'-variant RNA species (ΔNp73, ΔN'p73, ΔEx2p73, and ΔEx2/3p73) are translated into the amino-terminal truncated isoform, ΔNp73, which displays anti-apoptotic effects in tumor cells and primary neurons (Figure 1A) [14,15]. Importantly, several recent studies have demonstrated correlation between the relative expression of TA and/or ΔNp73 isoforms with patient survival for a variety of epithelial cancers occurring in adult patients [16-19].

Surveys have demonstrated overexpression of *TP73 *RNA in malignancies including colon and hepatocellular carcinoma, and neuroblastoma [20-22]. Neurodevelopmental pathways of clinical significance in neuroblastoma have also been implicated in medulloblastoma. A limited scale study failed to detect loss of heterozygosity or mutation of *TP73 *in five medulloblastoma samples [23]. However, to date no studies have examined the relative expression of TAp73 and ΔNp73 in medulloblastoma.

Here we report *TP73 *overexpression in a series of primary human medulloblastoma and in established medulloblastoma cell lines, relative to normal brain tissue. Kaplan-Meier analysis revealed trends toward favorable overall and progression-free survival of patients whose tumors display TAp73 RNA overexpression. Functional data indicate that overexpression of TAp73β and ΔNp73β induces apoptosis in transfected medulloblastoma cells with *wild-type TP53*. These results support the hypothesis that medulloblastoma express significant levels of p73 isoforms that can modulate survival. Our results support a role for *TP73 *in the growth of medulloblastoma and its response to genotoxic therapies.

## Methods

### Primary medulloblastoma specimens and medulloblastoma cell lines

Thirty-four medulloblastoma samples were obtained from children diagnosed between 1996 and 2004 at Texas Children's Hospital (Houston, TX) upon informed consent for an institutional review board-approved protocol (Table 1). All specimens were obtained at the time of diagnosis, snap-frozen and stored in liquid nitrogen. Histologic diagnoses were confirmed by pathologic review according to WHO criteria [12].

**Table 1:** Patient and tumor characteristics

***Patient and Tumor Characteristics***	***All Patients***	***Patients with Tumor TAp73 RNA < median***	***Patients with Tumor TAp73 RNA > median***
***Age at diagnosis ***(median)	80 months	79 months	79 months
< 36 months old (%)	4 (12)	2 (6)	2 (6)
> 36 months old (%)	30 (88)	15 (44)	15 (44)
***Sex***	**n (%)**	**n (%)**	**n (%)**
Male (%)	23 (68)	12 (35)	11 (32)
Female (%)	11 (32)	5 (15)	6 (18)
***Metastatic (Chang) Stage***	**n (%)**	**n (%)**	**n (%)**
M0, non-metastatic (%)	24 (71)	11 (32)	14 (41)
M1–M3, metastatic (%)	10 (29)	6 (18)	3 (9)
***Primary Resection Extent***	**n (%)**	**n (%)**	**n (%)**
Gross Total Resection (%)	23 (68)	12 (32)	11 (35)
Subtotal, Partial Resection or Biopsy only (%)	11 (32)	5 (15)	6 (18)
***Histologic Subtype***	**n (%)**	**n (%)**	**n (%)**
Classic, predominant (%)	8 (24)	4 (12)	3 (9)
Desmoplastic/Nodular, predominant (%)	12 (35)	6 (18)	7 (21)
Large Cell/Anaplastic, any features (%)	14 (41)	7 (21)	7 (21)
***Total***	**34 (100)**	**17 (50)**	**17 (50)**

Gross total resection was achieved in twenty-four patients. Chemotherapy for most patients consisted of cisplatin and vincristine, with combinations of carboplatin, etoposide, cyclophosphamide or lomustine. Sixteen patients received intensified chemotherapy with autologous stem cell support [24-27]. Patients greater than 36 months old received craniospinal irradiation 2400 ± 360 centiGray (cGy) with a tumor dose of 5300 ± 720 cGy. Median age at diagnosis was 78.9 months (range 12–216; mean 84 ± 4.3 months (± S.E.M.)) with median follow up of 42 months (range 4–88; mean 45 ± 3.9 months). For survival analysis, patients were stratified into two groups based on the median expression of TAp73 RNA in primary medulloblastoma tumor samples.

Established human medulloblastoma cell lines, Daoy and D283MED (D283), (American Type Culture Collection, Manassas, VA) were maintained in Dulbecco's modified Eagle's medium (Invitrogen, Carlsbad, CA) with high glucose (6 g/liter), 2 mM l-glutamine and 10% (vol/vol) heat-inactivated fetal calf serum (Invitrogen, Carlsbad, CA) at 37°C in 5% CO_2_. *TP53 *is mutated in Daoy and is *wild-type *in D283 cells [28,29]. For treatment studies, cells were exposed to etoposide (VP-16, 1–10 μM), cisplatin (CDDP, 5–25 μM), or vehicle (DMSO, 0.02%) control in complete media.

### Quantitative real-time RTPCR (qRT-RTPCR) analysis

Total cellular RNA was extracted with either TRIzol (Invitrogen, Carlsbad, CA) or RNeasy (Qiagen, Valencia, CA) based on tissue abundance, according to the manufacturers' recommendations. For reference controls, we used commercially available RNA from human fetal brain and human adult cerebellum (Stratagene, La Jolla, CA).   RNA integrity was verified on an Agilent 2100 Bioanalyzer (Agilent, Palo Alto, CA).

RNA was analyzed by qRT-RTPCR performed with the Bio-Rad iQ4 Multicolor Real-Time iCycler (Bio-Rad Laboratories, Hercules, CA). Total cellular RNA was reverse transcribed with Moloney murine leukemia virus reverse transcriptase (Invitrogen, Carlsbad, CA) and oligo-(dT)_12_, using standard methods. PCR reactions containing cDNA, iQ Syber Green Supermix (Bio-Rad Laboratories) and primers for *TP53*, full-length or 5'-terminal variant isoforms of *TP73 *were performed for 40 cycles in triplicate (Figure 1A). Specific primers detect RNA species for full-length human *TP73 *(TAp73) and 5'-terminal variants (ΔNp73, ΔN'p73, ΔEx2p73, and ΔEx2/3p73) (Figure 1A):

TAp73 sense, 5'-CCGGCGTGGGGAAGATGG-3' and

antisense, 5'-TTGAACTGGGCCATGACAGATG-3';

ΔNp73 (and ΔN'p73) sense, 5'-ACGGCCCAGTTCAATCTGC-3' and

antisense, 5'-CTGGGGTGTAGGGGCTGG-3';

ΔN'p73 sense, 5'-TCGACCTTCCCCAGTCAAGC-3' and

antisense, 5'-TGGGACGAGGCATGGATCTG-3';

ΔEx2p73 sense, 5'-AGGGAACCAGACAGCACCTA-3' and

antisense, 5'-ACGTCCATGCTGGAATCCG-3';

ΔEx2/3p73 sense, 5'-CAGGCCCAGTTCAATCTGCTG-3' and

antisense, 5'-GAGTGGGTGGGCACGCTG-3';

*TP53 *sense, 5'-CCATCTACAAGCAGTCACAGC-3' and

antisense, 5'-GAGTCTTCCAGTGTGAGATG-3';

*GAPDH *sense, 5'-AAGGTGAAGGTCGGAGTCAA-3' and

antisense, 5'-AATGAAGGGGTCATTGATGG-3'.

Because ΔNp73 lacks unique primer sequences, its relative abundance was calculated by subtracting from the relative copy number of ΔN'p73 (using the ΔN'p73-specific upstream primer pair) from those of ΔNp73 and ΔN'p73 determined using the common primer pair.

Amplification products were verified by agarose gel electrophoresis, melting curves, and sequencing. Gene expression was normalized internally to *GAPDH *expression, relative to control human fetal brain RNA as a tissue reference, and accounting for differences in primer efficiencies [30]. Results from at least two separate experiments were analyzed.

### Western immunoblot analysis

Cell lysates were prepared using standard methods. Briefly, protein was extracted from frozen tissue with boiling lysis buffer (0.5% SDS, 50 mM Tris-Cl, pH 8, 5 mM Na_2_EDTA) containing 2% β-mercaptoethanol for 5 minutes and tissue was homogenized using a PT1200CL Polytron (Kinematica, Switzerland), followed by shearing through a 22-guage needle and clarification by brief centrifugation. Yields were quantitated using the RC-DC Protein assay (Bio-Rad Laboratories, Hercules, CA). Lysates were separated by SDS-PAGE and transferred onto PVDF membranes for immunoblotting with antibodies against p73 (H-79, Santa Cruz Biotechnology, Santa Cruz, CA; and GC-15, Pharmingen, San Jose, CA), ΔNp73 (IMG-313, Imgenex, San Diego, CA), and p53 (FL-393 or DO-1, Santa Cruz Biotechnology), p21^Waf1 ^(Ab-11(CP74), Thermo Fisher Scientific, Fremont, CA), PARP (Cell Signaling Technology, Danvers, MA), and β-actin (C-2, Santa Cruz Biotechnology).

Chemiluminescent detection of primary antibody staining was performed using HRP-conjugated secondary antibodies (Jackson ImmunoResearch, West Grove, PA) with LumiGLO substrate, according to manufacturer's recommendations (Cell Signaling Technologies, Beverly, MA). Western blots were exposed to Kodak BioMax MS film (Kodak, New Haven, CT), developed, and analyzed with Image Station 2000R (Kodak). Alternatively, staining with fluorescent secondary antibody (Alexa Fluor 680 nm-conjugated goat anti-mouse IgG, Invitrogen) was visualized using an Odyssey infrared imaging system (LI-COR Biosciences, Lincoln, Nebraska). Intensity of immunostaining was quantitated using ImageQuant 5.2 (Molecular Dynamics, Piscataway, NJ). The intensity of each protein band was normalized to β-actin immunostaining as an internal loading control, and compared to results in the D283 cell line as a tissue reference. Results from at least two separate experiments were analyzed.

### Immunohistochemical analysis of primary human tumors

For immunostaining, five micron thick sections were prepared from formalin-fixed paraffin embedded medulloblastoma specimens. Tissue sections were deparaffinized in xylene, followed by graded hydration in 100% ethanol, 70% ethanol, and H_2_O. Antigen retrieval was performed by boiling in DAKO citrate for 25 min (Dako, Carpinteria, CA). Endogenous peroxidase was blocked with 3% H_2_0_2_/Methanol for 15 min, then incubated with 20% goat serum for 20 min. Sections were incubated with anti-p53 antibody (D07, Novocastra, Newcastle Upon Tyne, UK) for 1 hour at 25°C. This antibody is reactive for both wild-type and mutant p53 proteins. This was followed by incubation with HRP-conjugated goat anti-mouse secondary antibody for 30 min. Washed sections were developed using 3-amino-9-ethyl carbazole as the chromogen. The immunostained sections were graded semi-quantitatively for degrees of staining intensity (negative, 0; positive, 1+ to 3+) by neuropathologists blinded to clinical information.

### Gene expression and post-transcriptional gene silencing transfections

Daoy and D283 cell lines were transiently transfected with expression and reporter plasmids using Lipofectin according to manufacturer's recommendations (Invitrogen). Expression plasmids included: the human cytomegalovirus (CMV) immediate-early (IE) promoter-driven pP53-EGFP plasmid, encoding a p53-enhanced green fluorescent protein (GFP) fusion protein (Clontech, Mountainview, CA), and CMV IE promoter-driven hemaglutinnin-tagged human TAp73β- and Myc-tagged mouse ΔNp73β-encoding plasmids were previously described [14,31]. In each transfection, the expression plasmids were supplemented by empty vector (pcDNA3.1, Invitrogen) to achieve an equivalent total plasmid DNA concentration. For flow cytometry, the EGFP-expressing pmaxGFP plasmid (Amaxa, Gaithersburg, MD) provided transfection controls.

Transient small interfering RNA (siRNA)-mediated silencing of TAp73 and ΔNp73 was achieved using the following siRNAs: TAp73 (targeting a sequence in *TP73 *exon 3), 5'-AACGGAUUCCAGCAUGGACGU-3' [32]; ΔNp73 (targeting a sequence in *TP73 *exon 3B to exon 4), 5'-AACCUCGCCACGGCCCAGUUC-3'. Transfections with equimolar concentrations of validated siCONTROL non-targeting siRNA Pool#1 (Dharmacon, Lafayette, CO) provided negative controls. Daoy and D283 cells were grown to 50% confluency in a 6-well plate and transfected for 6 hours in Opti-MEM serum-free media (Invitrogen) with Oligofectamine Reagent, according to manufacturer recommendations (Invitrogen). Cells were allowed to recover from transfection for 24–48 hours and were then treated for 24 hours before harvesting for RNA or protein isolation. The resulting expression of target and off-target (e.g. β-actin and GAPDH) proteins were assayed by Western immunoblot analysis, relative to cells transfected with the negative control siRNA (Dharmacon).

### Apoptosis detection by flow cytometry and terminal deoxynucleotidyl transferase-mediated biotinylated-dUTP nick end-labeling (TUNEL)

We monitored DNA-indices for cell cycle analysis by multiparametric flow cytometry using standard methods. Analyses were performed using a Becton Dickinson FACScan flow cytometer (BD Biosciences, San Jose, CA) for the detection of cells stained with propidium iodide (PI) and a 488 nm laser with filter combination for fluorescein isothiocyanate (FITC) and GFP. Single cell suspensions were isolated from culture, fixed in methanol, and stained with PI (100 μg/mL in PBS). Each histogram represents 10,000–100,000 cells for measuring DNA-index and cell cycle. Histogram analysis was performed with the CellQuest program (BD Biosciences). We calculated the sub-G_0_/G_1 _peak in the hypodiploid distribution below a DNA index of one (< 2n). Because the nucleus becomes fragmented during apoptosis and numerous individual chromatin fragments may be present in a single cell, the percentage of objects with a fractional DNA-content is represented by the sub-G_0_/G_1 _peak. Apoptotic nuclei were identified as a hypodiploid DNA peak, and were distinguished from cell debris on the basis of forward light scatter and PI fluorescence.

To detect apoptotic nuclei in sub-G_0_/G_1 _peaks, a subset of unstained cells was analyzed for DNA-fragmentation using Frag-EL DNA fragmentation detection kit, according to manufacturer's recommendations (Oncogene Research Products, Cambridge, MA). Cells were also counter stained with PI for DNA quantification. FITC signal was detected on one channel in the logarithmic mode, while UV fluorescence (PI) was recorded in the linear mode on a separate channel. For each measurement, at least 10,000 cells were analyzed. We used CellQuest software for multiparametric calculations and analyses. Cut-off negative and positive cells resulted from FITC-fluorescence isotype control measurements for each sample.

### Statistical considerations

Clinical outcomes were analyzed for progression-free and overall survival using the method of Kaplan and Meier (StatView, v. 4.5, Abacus Concepts, Berkeley, CA; GraphPad Software, v.4; San Diego, CA). Patients were stratified according to tumor *TAp73 *RNA levels, normalized to *GAPDH *and relative to expression in fetal brain control RNA. The "high" expression group contained patients with tumor expression of TAp73 RNA greater than or equal to the median value of all tumors analyzed. The "low" expression group contained patients with tumor expression of TAp73 RNA less than the median value of all tumors analyzed. The significance of survival differences was calculated by logrank testing, which was applied to assess the significance of specific factors (e.g. patient stratification by *TP73 *expression level). To determine if expression levels correlated with other clinical and tumor characteristics, we applied Fisher's Exact testing.

## Results

### Medulloblastoma cell lines and primary medulloblastoma specimens overexpress *TP73 *RNA species relative to normal brain tissue

In order to examine the involvement of *TP73 *in medulloblastoma growth, we surveyed expression levels of TAp73 and total ΔNp73 variants (ΔNp73, ΔN'p73, ΔEx2p73, and ΔEx2/3p73) using qRT-RTPCR. Compared to human fetal brain, Daoy and D283 cell lines, as well as adult human cerebellum overexpressed TAp73 and total ΔNp73 RNA variants, internally normalized to *GAPDH *(Figure 1B). *TP53*-mutant Daoy cells displayed much higher levels of TAp73 and of total ΔNp73 RNA (9-fold ± 1 (mean ± S.E.M.) and 12-fold ± 2, respectively) than fetal brain. D283 cells expressed even higher levels of TAp73 and total ΔNp73 (116-fold ± 35 and 74-fold ± 16, respectively).

Since established cell lines *in vitro *differ significantly from medulloblastomas *in vivo*, we analyzed primary tumor specimens from 34 patients. By qRT-RTPCR, the RNA expression of TAp73 and total ΔNp73 variants was similar among primary medulloblastoma samples but markedly higher than cell lines (Figure 1B–C, see Additional file 1). Primary tumors displayed 282-fold (± 65) more TAp73 and 194-fold (± 36) more total ΔNp73 RNA than human fetal brain.

To provide an additional tissue control, we analyzed *TP73 *5'-variants in RNA from human adult cerebellum. Compared to human fetal brain, adult cerebellum also displayed overexpression of TAp73 and total ΔNp73 RNA (24-fold ± 6 and 48-fold ± 5, respectively) (Figure 1B). Importantly, primary medulloblastoma samples expressed about 10-fold more *TP73 *RNA than normal adult cerebellum (Figure 1B). Of the four control tissue controls, male and female fetal brain RNA (Stratagene) revealed similar *TP73 *expression profiles. As shown in Figure 1B, adult cerebellum expressed 10 to 100-fold more TAp73 and ΔNp73 RNA compared to human fetal brain controls. We also examined *TP73 *RNA expression in human adult frontal cortex (Stratagene) by qRT-RTPCR, but found minimal expression of TAp73 or ΔNp73 transcripts (data not shown).

Since the effects of p73 isoforms likely reflect their ability to interact with p53, we assessed *TP53 *expression using primers in exons 5 and 7 to detect all known *TP53 *splice variants (Figure 1B, see Additional file 1). The Daoy cell line, which harbors a p53 mutation (C242F), expressed *TP53 *RNA at a level similar to human fetal brain, 1.1-fold (± 0.42), but 15-fold (± 2.0) more than human adult cerebellum. The D283 cell line expressed *TP53 *RNA at much lower levels than human fetal brain, 0.05-fold (± 0.016) and 0.43-fold (± 0.14) relative to human adult cerebellum. Primary medulloblastoma specimens displayed *TP53 *levels 1.4-fold (± 0.12) higher than in human fetal brain, and 12-fold (± 1.0) higher than in human adult cerebellum (Figure 1B). *TP53 *copy numbers in cell lines were at least one log lower than either TAp73 or total ΔNp73 levels, whereas in primary tumors *TP53 *was at least two logs lower than either TAp73 or total ΔNp73.

### Primary tumors and cell lines overexpress TAp73 and ΔNp73 proteins

**Figure 2:**
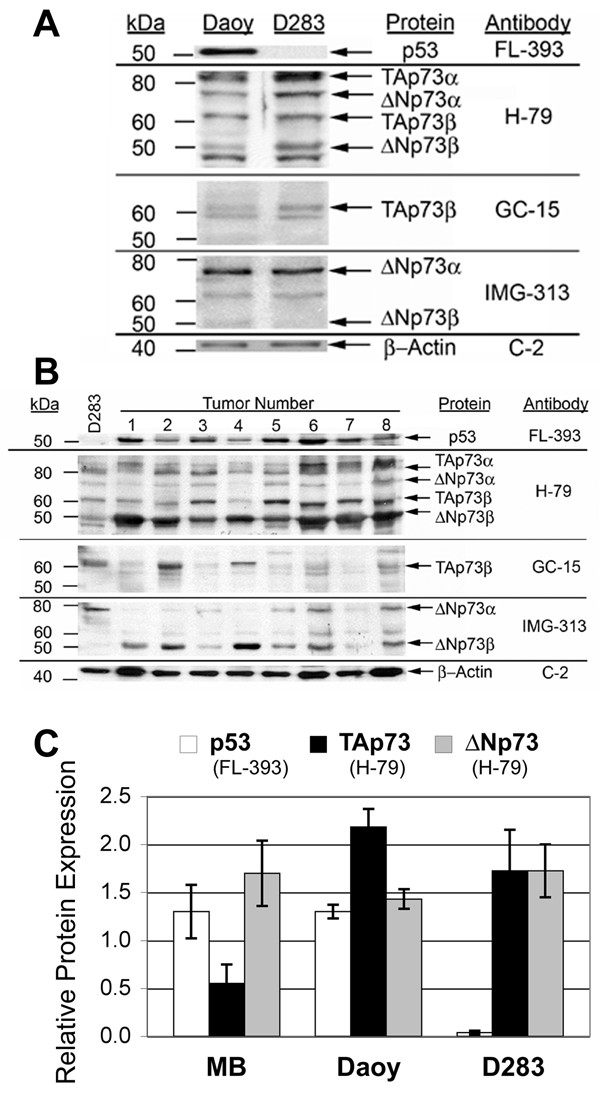
**Primary medulloblastoma specimens and medulloblastoma cell lines overexpress TAp73 and ΔNp73 proteins**. Western immunoblot analysis of p53, TAp73, and ΔNp73 protein expression in: **(A) **established medulloblastoma cell lines, Daoy and D283MED (D283), and **(B) **primary medulloblastoma specimens. Immunoblots for p53 (FL-393) or p73 proteins (H-79, GC-15, and IMG-313) were stripped and re-probed for β-actin (C-2) to control for protein loading. Columns marked with numbers 1–8 represent Western blots of eight different primary medulloblastoma specimens. Additional bands presumably represent additional carboxy-terminal isoforms of TAp73 and ΔNp73. Relative migration of molecular weight standards is shown at left. Shown are representative blots from at least 3 experiments, normalized to ß-actin (mean ± S.E.M.).   **(C) **Primary medulloblastoma (MB) specimens and medulloblastoma cell lines display significant expression of TAp73, ΔNp73, and p53 proteins as quantitated on Western blots. Shown are representative blots from at least 3 experiments, normalized to ß-actin (mean ± S.E.M.).

In order to examine expression of p73 proteins in established cell lines and primary medulloblastoma samples, we employed monoclonal (GC-15) and polyclonal (H-79) antibodies, which recognize full-length TAp73 and amino-truncated ΔNp73 [33], and a ΔNp73-specific monoclonal antibody (IMG-313) (Figure 2A–B, see Additional file 2). Protein levels were internally normalized to β-actin expression as a loading control and compared to expression in the D283 cell line as a reference.

The H-79 polyclonal antibody, raised against a conserved amino-terminal peptide sequence, recognizes the major carboxy-terminal variants of TAp73 (TAp73α, 75–80 kDa; and TAp73β, 60–65 kDa) and of ΔNp73 (ΔNp73α, 70–75 kDa; and ΔNp73β, 50 kDa) [34]. Both established medulloblastoma cell lines, Daoy and D283, exhibited significant, although different levels of TAp73 and ΔNp73, relative to β-actin (Figure 2C). Primary tumor specimens (n = 8), in contrast, exhibited more ΔNp73 (1.7-fold D283 levels (± 0.34)) compared to TAp73 (0.55-fold ± 0.2).

The GC-15 monoclonal antibody, raised against a conserved carboxy-terminal peptide sequence, found in p73β isoforms, also detected comparable levels of TAp73 protein in established cell lines and in primary medulloblastoma samples (Figure 2A–B). To confirm the identity of putative ΔNp73 bands, we used the ΔNp73-specific IMG-313 antibody (Figure 2A–B). Based on their predicted molecular weights, the other detected isoforms presumably represent other carboxy-terminal variants as reported by others [35]. The equivalent expression of TAp73 and ΔNp73 protein in D283 cells resembled its RNA expression pattern by qRT-RTPCR. In contrast, Daoy cells expressed slightly more TAp73 than ΔNp73 protein, and primary tumor samples displayed higher levels of ΔNp73 compared to TAp73 protein.

Primary medulloblastoma specimens and cell lines displayed variable p53 protein levels (Figure 2A–B, see Additional file 3). Daoy cells express high levels of mutant p53. The higher p53 level in Daoy cells suggests decreased turnover by MDM2-mediated degradation as commonly seen with many mutant p53 proteins. D283, which is *wild-type *for *TP53*, expresses much lower levels of p53 protein at baseline (Figure 2C). The relative p53 protein levels in established cell lines and primary medulloblastoma are similar to their pattern of *TP53 *RNA expression. Using antibodies with differing affinities precluded a direct comparison of p53 and p73 protein levels.

**Figure 3:**
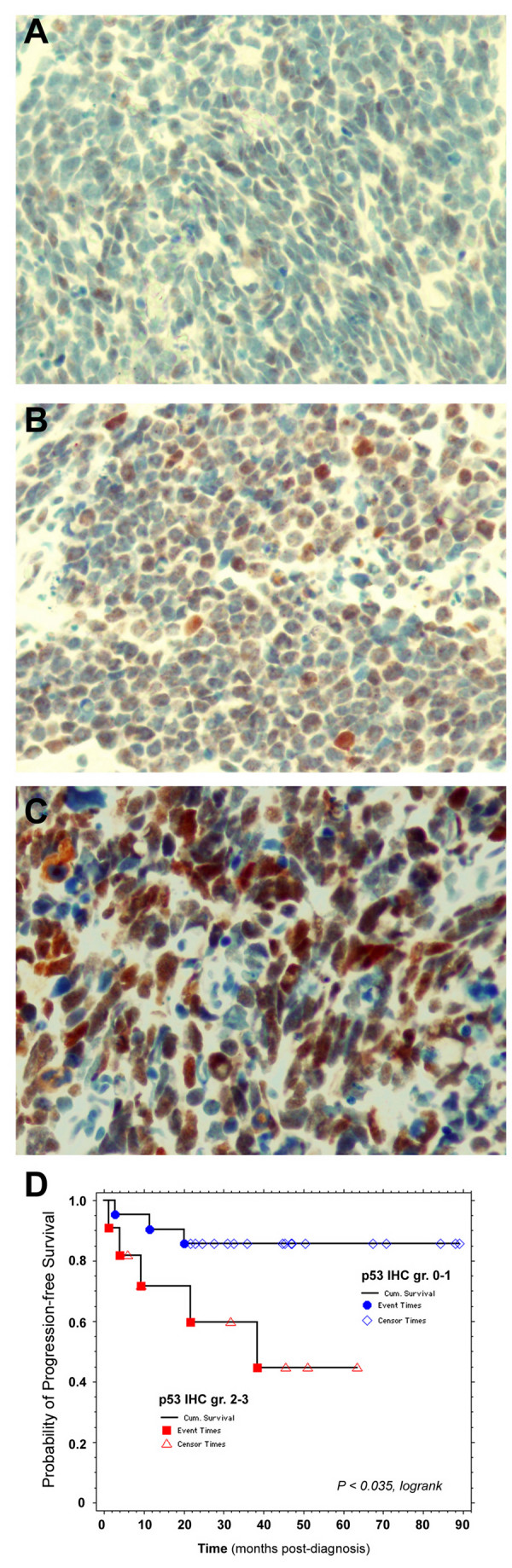
**Intensity of p53 immunostaining correlates with overall survival**. Examples of medulloblastoma sections displaying **(A) **1+, **(B) **2+, and **(C) **3+ intensity of p53 immunostaining. **(D) **Kaplan-Meier survival analysis illustrating association of 2+ and 3+ intensity with adverse progression-free survival (p < 0.035 by logrank testing)**.**

Although *TP53 *RNA expression did not correlate with clinical outcome, immunohistochemical staining intensity of p53 protein (scored > 2+) in paraffin-embedded formalin-fixed specimens was significantly associated with worse progression-free survival (PFS) (p < 0.035, Figure 3). These findings underscore the central importance of p53, which can interact with various p73 isoforms.

### Overall and progression-free survival are associated with TAp73 overexpression

**Figure 4:**
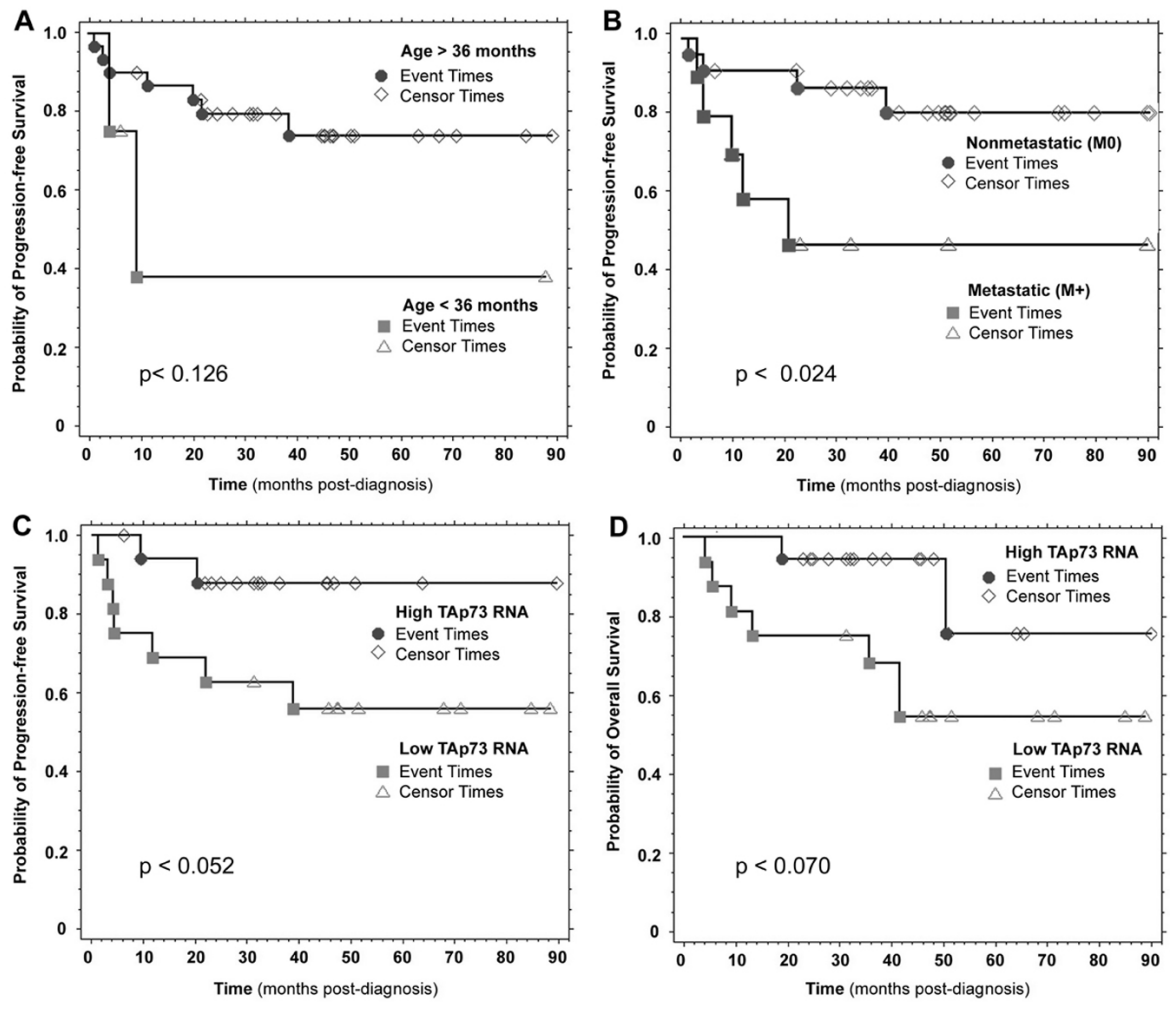
**Medulloblastoma survival is associated with TAp73 overexpression**. Kaplan-Meier analysis reveals trends toward adverse outcome associated with **(A)**  age less than 36 months (p < 0.126 by logrank), and  **(B)**  metastatic (M+) disease at diagnosis (p < 0.024); while revealing  **(C)**  a trend toward better PFS (p < 0.052) and **(D)**  better OS and in patients whose tumors display higher TAp73 RNA levels (p < 0.070). Patients were stratified (**High **vs. **Low**) according to tumor TAp73 levels, normalized to *GAPDH *and relative to fetal brain control. **High **= greater than or equal to the median tumor expression of TAp73 RNA. **Low **= less than the median tumor expression of TAp73 RNA.

For the entire group of patients studied, the median PFS was 35.4 months (range 4.0 – 88.3; 38.1 ± 4.5 months (mean ± S.E.M.)). We analyzed outcome among subsets of patients with respect to clinical variables such as age, extent of primary resection, or metastatic disease at diagnosis. As established in other series, those children less than 36 months old (p < 0.160 and p < 0.126 for overall survival (OS) and progression-free survival (PFS) by logrank, respectively) or with metastatic disease (M-positive Chang stage; p < 0.044 (OS) and p < 0.024 (PFS)) displayed a trend toward worse OS and PFS, but did not achieve statistical significance (Figure 4A–B). OS and PFS were not significantly associated with patient sex, degree of initial resection, or chemotherapy regimen.

The association of *TP73 *expression with clinical outcome in neuroblastoma, the most common malignant solid tumor of childhood, prompted our analysis of the relationship between *TP73 *RNA and survival in medulloblastoma patients. When stratified by tumor TAp73 RNA levels, higher expression was associated with better PFS (p < 0.052) and OS (p < 0.070) (Table 1; Figure 4C–D). We also examined RNA expression of *TP53*, TAp73, or ΔNp73 variants, and histologic subtype, but found no statistically significant association with OS or PFS. *TP53 *and *TP73 *RNA levels did not correlate with other prognostic factors such as patient age, metastatic status, or extent of resection, with p values ranging from 0.35 to 1.00 (Fisher's Exact test).

### Genotoxic stress induces *TP73 *expression in medulloblastoma cell lines

**Figure 5:**
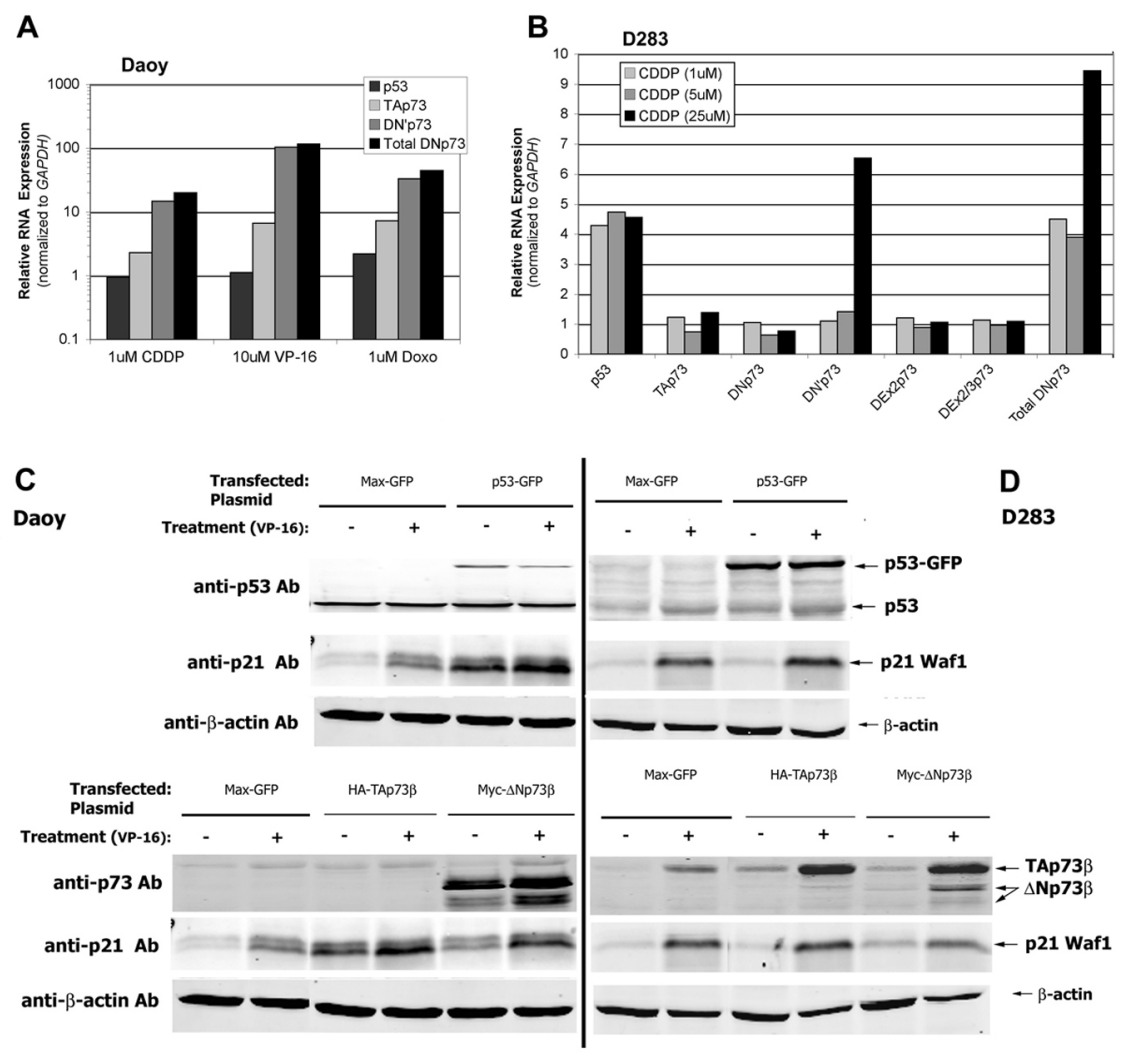
**Genotoxic agents induce *TP73 *expression and apoptosis in medulloblastoma cell lines**. **(A)  **Chemotherapeutic agents (cisplatin **(CDDP)** 1 µM, etoposide **(VP-16) **10 µM, and doxorubicin **(DOXO)** 1 µM) induce *TP73* expression in Daoy cells, especially ΔN’p73 species, as determined by qRT-RTPCR normalized to GAPDH expression. ** (B)**  CDDP-treated (1, 5, and 25 µM) D283 cells display induction of *TP53* and *TP73* RNA expression (including ΔN’p73 species) - similar to results observed in CDDP-treated Daoy cells. Western immunoblots reveal that** (C) **Daoy cells and **(D)**  D283 cells transiently transfected with p53-GFP expression plasmid increase expression of tagged p53-GFP protein (at a higher apparent molecular weight than native p53), as well as the p53/p73 target, p21^Waf1^**(arrows, upper panels)**. Treatment with etoposide (VP-16, 1.5 µM) stabilizes wild-type p53 protein.  Transient transfection with either TAp73 or ΔNp73 expression plasmids also increased respective protein levels, normalized to ß-actin loading control. **(arrows lower panels).  **

Because the analyzed tumor specimens were obtained before genotoxic treatment, gene expression levels do not reflect the activity of p53 and p73 isoforms. Therefore, we used medulloblastoma cell lines to assay responses to genotoxic stress in the form of chemotherapeutic agents used to treat medulloblastoma clinically (cisplatin (CDDP), etoposide (VP-16)) and another agent, doxorubicin, previously studied for *TP73 *induction (Figure 5A). When exposed to cytotoxic concentrations of these agents, *TP53 *and *TP73 *RNA levels, especially the ΔN'p73 species, increased in D283 cells (Figure 5B). Although less p53 induction was observed in *TP53 *mutant Daoy cells, increased *TP73 *levels including ΔN'p73 were detected (Figure 5C). These data indicate that in addition to post-translational stabilization, p73 levels are regulated at least in part through chemotherapy-induced p73 RNA transcription.

D283 and Daoy cells were also used for transfection studies to determine the effects of overexpressed TAp73 and ΔNp73. Treatment with VP-16 stabilized p53 levels in D283 cells (Figure 5D). We examined the effects of increased p53, TAp73, and ΔNp73 levels by transient transfection with expression plasmids. Transient transfected p53, TAp73, and ΔNp73 could be readily distinguished on Western blots of D283 and Daoy cell lines (Figure 5C–D).

### Chemotherapy and overexpressed p53 induce apoptosis

We investigated whether p53 induces apoptosis in medulloblastoma cells exposed to chemotherapeutic agents. Expression plasmid transfections also permitted flow cytometric analysis of the cell cycle distribution of fluorescently immunolabeled TAp73β-, ΔNp73β-, and p53-overexpressing transfectants. VP-16-induced changes in apoptosis were determined by flow cytometric quantitation of cells in sub-G_0_/G_1 _fractions, which was confirmed by TUNEL analysis (see Additional file 4). Similar results were obtained with CDDP and UV irradiation (data not shown).

**Figure 6:**
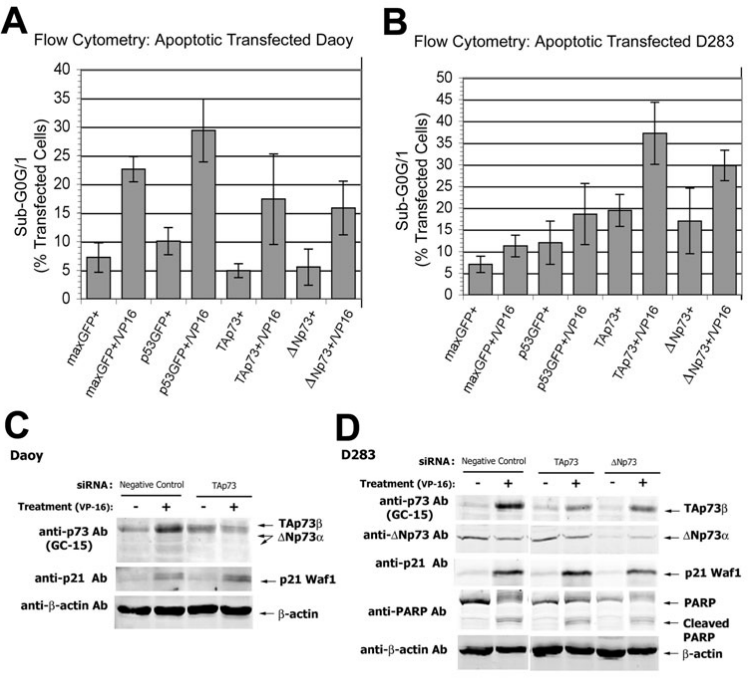
**TAp73 induces apoptosis of medulloblastoma cell lines**. Histograms summarizing flow cytometric analysis of cell cycle distributions of: **(A)** transfected Daoy cell line, illustrating the sub-G0/G1 peak representing apoptotic nuclei; and **(B)**  transfected D283 cell line in sub-G0/G1, with plasmids and treatment with VP-16 (1.5 µM), as indicated.  Y-axis, % of cells with apoptotic features by flow cytometry; X-axis, transfected plasmid(s) and culture conditions. Western immunoblots also reveal that ** (C)** Daoy cells and ** (D)** D283 cells transiently transfected with isoform-specific siRNA reveal knockdown of their respective protein levels.  D283 knockdowns reduced protein expression of the p53/p73 target, p21^Waf1^, and cleavage of PARP **(arrows)**.

*TP53 *mutant Daoy cells treated with VP-16 increased their apoptotic sub-G_0_/G_1 _population to 22.6% compared to 7.2% in untreated controls, illustrating VP-16 induced apoptosis in the absence of active p53 (Figure 6A). Overexpression of transfected *wild-type *p53 in Daoy cells caused increased apoptosis (10.1% in sub-G_0_/G_1_), compared to controls (7.2%) (Figure 6A). VP-16 treatment of p53-expressing Daoy transfectants further increased apoptosis (29.4%) (Figure 6A). These data indicate that transfected p53 expression partially restored p53 function in *TP53*-mutant Daoy cells.

When exposed to VP-16, D283 controls (transfected with a GFP-expressing control plasmid) underwent increased apoptosis as indicated by an increased fraction of cells in sub-G_0_/G_1_, from 7.0 to 11.3%, (Figure 6B). These results indicate that VP-16 induces apoptosis, as described for other neuroepithelial cell types with *wild-type *p53. Overproduction of p53 in D283 cells by transfection of a GFP-tagged p53 expression plasmid increased apoptosis (12.0% in sub-G_0_/G_1_) compared to controls (7.0%) (Figure 6B). VP-16 treatment of p53-transfected D283 cells further increased the sub-G_0_/G_1 _population to 18.6% (Figure 6B).

Overexpression of p53 in transfected Daoy cells increased expression of p21^Waf1 ^protein, relative to control maxGFP-transfected cells (**upper panels**, Figure 5C). VP-16 treatment of p53-overexpressing D283 cells also increased p21^Waf1 ^protein demonstrating the activity of transfected p53 (**upper panels**, Figure 5D).

### Overexpression of TAp73β or ΔNp73β induces apoptosis

We examined the effects of TAp73 overexpression on survival. TAp73β overexpression increased apoptosis to 19.4% from 7.0% in GFP-control transfected *wild-type TP53 *D283 cells, even in the absence of genotoxic stress. VP-16 treatment of TAp73β transfectants resulting in further enhancement of chemosensitivity with 37.2% in sub-G_0_/G_1 _compared to 11.3% treated control and 19.4% in untreated TAp73β transfectants (Figure 6). Transfected TAp73β induced apoptosis as effectively as transfected p53 (Figure 6A). The sub-G_0_/G_1 _population in p53 mutant Daoy cells transfected with TAp73β did not differ significantly from control transfectants under basal conditions (Figure 6B).

We next asked whether ΔNp73 affects survival in medulloblastoma cells. Rather than anti-apoptotic effects, ΔNp73β overexpression increases apoptosis to 17.0% compared to 7.0% in GFP-control transfected D283 (Figure 6A). Transfection of ΔNp73β in D283 also increased apoptosis in response to VP-16-treated D283 (29.8% in sub-G_0_/G_1_) (Figure 6A). Neither of these effects was observed in transfected p53 mutant Daoy cells. These data suggest that apoptosis and chemosensitization induced by TAp73β and ΔNp73β, as in D283 cells, require *wild-type *p53.

As noted with p53 overexpression, transient transfection with expression plasmids encoding TAp73β or ΔNp73β resulted in increased protein expression of the p53/p73 target gene p21^Waf1^. Overexpression of TAp73β resulted in a comparable increase of p21^Waf1 ^expression in Daoy cells and untreated D283 cells, relative to control transfectants (**lower panels**, Figure 5C–D). Overexpression of ΔNp73β also increased p21^Waf1 ^protein expression (**lower panels**, Figure 5C–D). Treatment of p53-, TAp73β – and ΔNp73β-transfected D283 cells with VP-16 revealed similar induction of p21^Waf1 ^(Figure 5D). These results demonstrate the transcriptional activity of overexpressed *TP73 *isoforms in medulloblastoma cell lines.

### Knockdown of TAp73 or ΔNp73 reduces target gene expression and apoptosis

Transient transfection of Daoy and D283 cells with isoform-specific siRNAs decreased TAp73 and ΔNp73 protein expression by Western blot analysis (Figure 6C–D). In D283 transfectants, TAp73 siRNA decreased the protein expression of the p53/p73 target gene p21^Waf1 ^(Figure 6D). As an indicator of decreased apoptosis, PARP cleavage was also decreased by TAp73 knockdown in D283 (Figure 6D). In contrast, D283 cells transfected with ΔNp73 siRNA did not display p73 target gene induction or PARP cleavage in D283 (Figure 6D). Reducing ΔNp73 expression siRNA resulted in decreased p21^Waf1 ^protein as seen in TAp73 knockdowns (Figure 6D). These knockdown data complement the results of overexpression and apoptosis assays.

## Discussion

We have determined that primary medulloblastoma specimens and cell lines overexpress full-length TAp73 and the amino-terminal truncated variants of *TP73 *(ΔNp73, ΔN'p73, ΔEx2p73, and ΔEx2/3p73) at levels in excess of those found in fetal brain and normal adult cerebellum. We have also shown that adult cerebellum express more TAp73 and 5'-terminal variant RNAs than human fetal brain. This is consistent with results from studies in murine brain showing that the relative balance of *TP73 *expression appears to shift during development with increasing overall levels [14]. Normal neuronal differentiation requires a balance between TAp73 and ΔNp73 [14,15]. While ΔNp73 is necessary for adult neuronal survival, levels in excess of these physiologic anti-apoptotic levels may prevent normal cell death during development, following growth factor withdrawal, or in response to genotoxic stress such as chemotherapeutic agents [14].

Studies of *TP73 *expression in mature CNS have been limited. In adult mouse brain, *TP73 *is most highly expressed in the cerebellum [36]. Other studies of murine models have described the crucial role of ΔNp73 in survival of neurons in the CNS [37,38]. In contrast, *TP73 *expression and function in normal human brain has not been fully characterized. Others have reported higher *TP73 *expression in human tumor tissue than in normal brain tissue [39-41]. While human fetal brain reportedly expresses high levels of ΔNp73 in Cajal-Retzius cells [42], adult cortical and hippocampal neurons apparently express significant levels of both TAp73 and ΔNp73 [43]. While human cerebellum has not been previously studied, our qRT-RTPCR results confirm *TP73 *expression in non-neoplastic CNS tissue. Aberrant *TP73 *expression in the cerebellum may divert developing neurons from normal differentiation, leading to uncontrolled proliferation and medulloblastoma formation.

We have also confirmed high levels of expression of TAp73 and ΔNp73 proteins in the same tumors and cell lines. Among different cell types, the apparent discrepancy between expression of *TP73 *variants at the RNA and protein levels suggests that post-translational modifications also contribute to observed levels. Like other transcription factors, *TP73 *variants are regulated by a variety of positive and negative networks involving: E2F1, MDM2, Sumo-1, c-Abl, NEDL2, and other DNA damage response factors [21,44]. Such complex regulatory influences probably account for the dynamic expression levels of *TP73*, as seen with p53.

Our analysis of primary medulloblastoma was limited to those specimens with sufficient quantities available as freshly snap frozen tissue suitable for analysis. This fact may represent an unintended selection bias. However, more importantly, none of our findings correlates with histologic subtype or clinical features. Nonetheless, we detected trends toward favorable overall and progression-free survival of patients whose tumors display higher levels of TAp73 RNA, similar to reports in certain adult tumor types [16-19,39]. Future inclusion of additional patients and longer follow-up may increase the strength of the association between *TP73 *and outcome.

*TP73 *overexpression in medulloblastoma samples compared to normal brain provides potential evidence for p73 isoforms in the growth and treatment response of medulloblastoma. In contrast to normal brain tissue, primary tumor specimens and established medulloblastoma cell lines overexpress both TAp73 and ΔNp73 proteins, consistent with dysregulation of developmental expression and a possible contribution to their neoplastic phenotype. Functional studies of TAp73 and ΔNp73 have revealed differential effects in response to genotoxic stress [16,44-46]. TAp73 induces arrest and apoptosis, while in most systems ΔNp73 counteracts these pathways in response to ionizing radiation and genotoxic agents *in vitro *[as reviewed in [47]]. In other cell types, ΔNp73 induces apoptosis instead, underscoring the complexity of p73 isoform function. The frequency and degree of *TP73 *overexpression in primary medulloblastoma strongly suggests the acquisition of selective advantage(s). Higher relative levels of TAp73 may enhance the apoptotic response of medulloblastoma.

Since p73 isoforms interact with p53 and can activate common target genes, overexpression of TAp73 and ΔNp73 may affect medulloblastoma growth via interaction with *TP53 *and may have functional significance in medulloblastoma. The association between apparent p53 overexpression and clinical outcome in medulloblastoma may not reflect the effects of *wild-type *p53. Since mutant or dominant negative p53 isoforms are also detected by the nonselective antibodies used, conclusions regarding potential interactions between p53 and p73 cannot be drawn in the absence of *TP53 *sequencing.

Etoposide and cisplatin are among the chemotherapeutic drugs important in the treatment of medulloblastoma, and known to activate TAp73. Cisplatin reportedly increases p73 levels by post-translational stabilization, but we detected increased p73 RNA species, most notably the ΔN'p73 variant, suggesting a component of transcriptional induction. Our results indicate that chemotherapy-induced genotoxic stress induces apoptosis in D283 and Daoy cells. Our results are consistent with studies of other *wild-type *p53 cell types, including neuroepithelial cells, indicating that VP-16 induces apoptosis in a p53-related manner [48-50]. Furthermore, p53 overexpression alone induces apoptotic cell death, indicating that overexpression of p53 can overcome endogenous negative regulation in D283 cells and *TP53*-mutation in Daoy cells.

To address the functional significance of TAp73 and ΔNp73 overexpression, we transiently transfected medulloblastoma cell lines and examined their survival. Medulloblastoma cell lines with *wild-type *and mutant *TP53 *displayed distinct effects of *TP73 *overexpression. In addition to high levels of basal TAp73 and ΔNp73 in D283, we found that exogenous TAp73β and ΔNp73β increased apoptosis, especially in response to VP-16. In *TP53 *mutant Daoy cells, overexpressed TAp73β and ΔNp73β had little effect, even in response to VP-16.

In general, overexpression of p53, TAp73β, and ΔNp73β each induced expression of p53/p73 target genes associated with arrest and apoptosis. As expected, knockdown of p53 or TAp73 was associated with reduced expression of these proteins. These results support our TUNEL and flow cytometric data, providing evidence that modulation of p53 and p73 functions can influence chemosensitivity in medulloblastoma. These data suggest that TAp73 regulates apoptosis in medulloblastoma, but is dependent on the p53 status. One possible explanation for this effect is that some forms of mutant p53 can hetero-oligomerize with and inactivate TAp73 [33,51]. Thus, transfected TAp73 may be sequestered in inactive oligomers with mutant p53 preventing activation of p73 pro-apoptotic target genes. In addition to direct interaction with p53, p73 isoforms co-regulate shared downstream transcriptional targets of p53.

The initial depiction of ΔNp73 and TAp73 as either anti- or pro-apoptotic appears oversimplified [as reviewed in [47]]. In fact, various chemotherapeutic agents can induce ΔNp73 expression in neoplastic cell lines, suggesting a role in apoptotic response [52,53]. Others have noted that constitutive expression of ΔNp73 isoforms can either resist or increase apoptosis while inducing p53 target gene expression [54,55]. The pro-apoptotic effects of ΔNp73 have been previously described and may reflect cell type-dependent factors other than specific interactions with p53. These emerging data indicate that the diversity of p73 functions depend upon the cellular context so that the pattern of isoforms expressed may cause pro- or anti-apoptotic effects. These factors add further to the complexity of interpreting the chemosensitizing effects and overabundance of p73 isoforms that we have observed in medulloblastoma. Nonetheless, our results indicate that TAp73 and ΔNp73 are overexpressed in primary medulloblastoma and demonstrate pro-apoptotic effects similar to those of p53, which may provide mechanisms for modulating tumor cell survival and clinically relevant prognostic information.

## Conclusion

Our analysis of 34 medulloblastoma patient specimens and the medulloblastoma cell lines Daoy and D283 demonstrates significant expression of full-length, TAp73, and amino-terminal-truncated, ΔNp73, *TP73 *RNA transcripts and protein. Although the correlation between *TP73 *RNA expression and patient survival did not achieve statistical significance, there was a trend toward improved overall and progression-free survival in patients whose tumors exhibited high expression of TAp73 RNA. Furthermore, by overexpressing or knocking-down TAp73 or ΔNp73 expression, we have implicated *TP73 *isoforms in chemoresponsiveness of medulloblastoma. These results suggest that expression of the *TP73 *isoform TAp73 in medulloblastomas may be useful as a prognostic marker for patient survival. Our results also suggest that therapeutic strategies that increase *TP73 *expression may be useful to augment responsiveness of medulloblastomas to chemotherapy.

## List of abbreviations

qRT-RTPCR, quantitative real-time reverse transcriptase polymerase chain reaction; TUNEL, terminal deoxynucleotidyl transferase-mediated biotinylated-dUTP nick end-labeling; OS, overall survival; PFS, progression-free survival; CDDP, cisplatin; VP-16, etoposide; DOXO, doxorubicin.

## Competing interests

The author(s) declare that they have no competing interests.

## Authors' contributions

RCC, MDB, LLL, JAR, DGS, MSI, and JYHK contributed to expression and functional analyses. RCC, MDB, LP, AMA, MSI, and JYHK analyzed primary tumor pathology, clinical characteristics and patient outcomes. RCC, MDB, and JYHK performed the statistical analyses. AMA, RCC, and JYHK analyzed immunohistochemistry of tumor tissue sections. MSI and JYHK conceived the study, and with RCC and MDB participated in its design, coordination, and manuscript composition. All of the authors made significant contributions to data interpretation, and have read and approved the final manuscript.

## Pre-publication history

The pre-publication history for this paper can be accessed here:



## Supplementary Material

Additional file 1 ***TP73 *RNA expression in primary medulloblastoma samples**. Individual primary medulloblastoma samples from patients (n = 34) display overexpression of TAp73 (TA) and 5'-terminal variant RNA species, relative to human fetal brain and normalized to *GAPDH *expression. By comparison, *TP53 *RNA is relatively underexpressed. Total ΔNp73 represents the sum of expression of all 5'-terminal variant *TP73 *RNA variants (ΔNp73, ΔN'p73, ΔEx2p73, and ΔEx2/3p73). ***Columns***, mean expression of at least 2 experiments; ***error bars***, ± S.E.M. ***Y-axis***, RNA expression relative to human fetal brain and normalized to *GAPDH *expression (N.B. log-scale). Click here for file

Additional file 2 **Primary medulloblastoma samples express p53 protein**. Western blot analysis reveals that individual primary medulloblastoma samples from patients express p53 (internally normalized to β-actin expression and relative to D283 cells). Click here for file

Additional file 3 **TAp73 and ΔNp73 protein expression in primary medulloblastoma samples**. Western blot analysis reveals that individual primary medulloblastoma samples from patients display overexpression of full-length TAp73 and amino-terminal truncated ΔNp73 (internally normalized to β-actin expression and relative to D283 cells). Click here for file

Additional file 4 **Apoptosis analysis**. TUNEL assay of D283 cell line confirms apoptotic changes in PI-stained cell populations in the sub-G_0_/G_1 _peak (i.e. hypodiploid (less than 2n) DNA content, representing apoptotic nuclei) detected using flow cytometric methods. ***X-axis***, transfected plasmids (described in text) and post-transfection culture conditions (VP-16, 1.5 μM); ***Y-axis***, % of cells with apoptotic features by flow cytometry or TUNEL assay. Click here for file
