# Supplementary figures and images for: Overexpressed TP73 induces apoptosis in medulloblastoma
Source: BMC Cancer. 2007 Jul 12;7:127. doi: 10.1186/1471-2407-7-127 (PMC1955450; doi:10.1186/1471-2407-7-127)

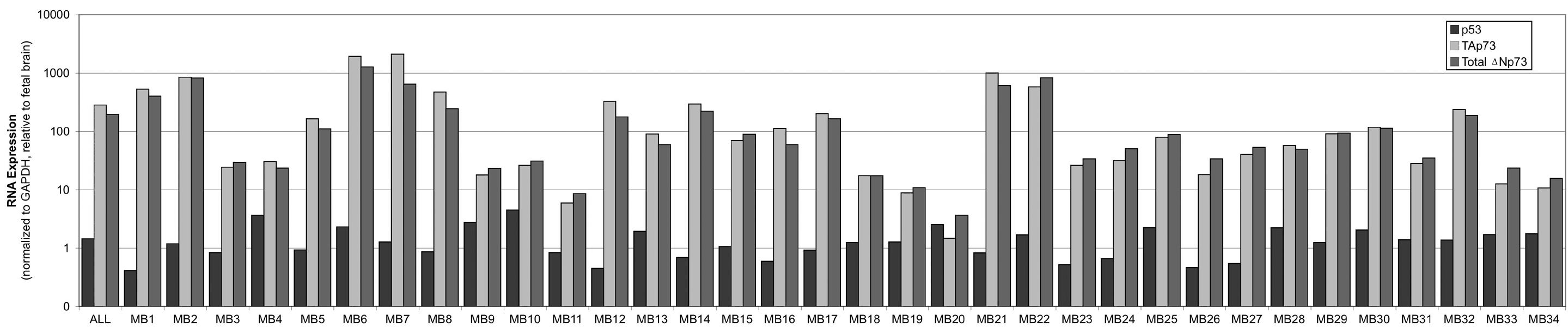

Supplement: Additional file 1 — TP73 RNA expression in primary medulloblastoma samples. Individual primary medulloblastoma samples from patients (n = 34) display overexpression of TAp73 (TA) and 5'-terminal variant RNA species, relative to human fetal brain and normalized to GAPDH expression. By comparison, TP53 RNA is relatively underexpressed. Total ΔNp73 represents the sum of expression of all 5'-terminal variant TP73 RNA variants (ΔNp73, ΔN'p73, ΔEx2p73, and ΔEx2/3p73). Columns, mean expression of at least 2 experiments; error bars, ± S.E.M. Y-axis, RNA expression relative to human fetal brain and normalized to GAPDH expression (N.B. log-scale). [file 1471-2407-7-127-S1.jpeg]

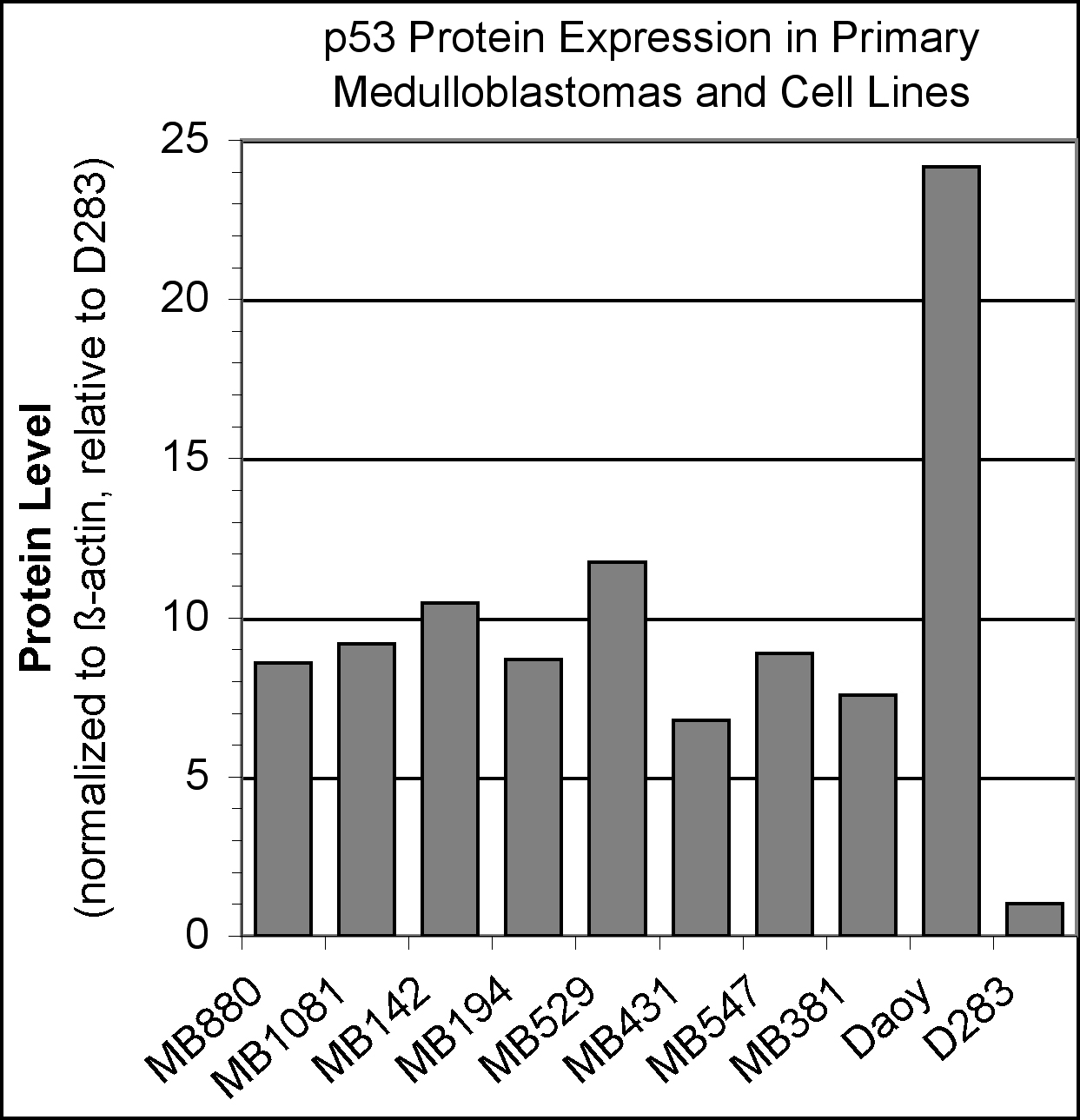

Supplement: Additional file 2 — Primary medulloblastoma samples express p53 protein. Western blot analysis reveals that individual primary medulloblastoma samples from patients express p53 (internally normalized to β-actin expression and relative to D283 cells). [file 1471-2407-7-127-S2.jpeg]

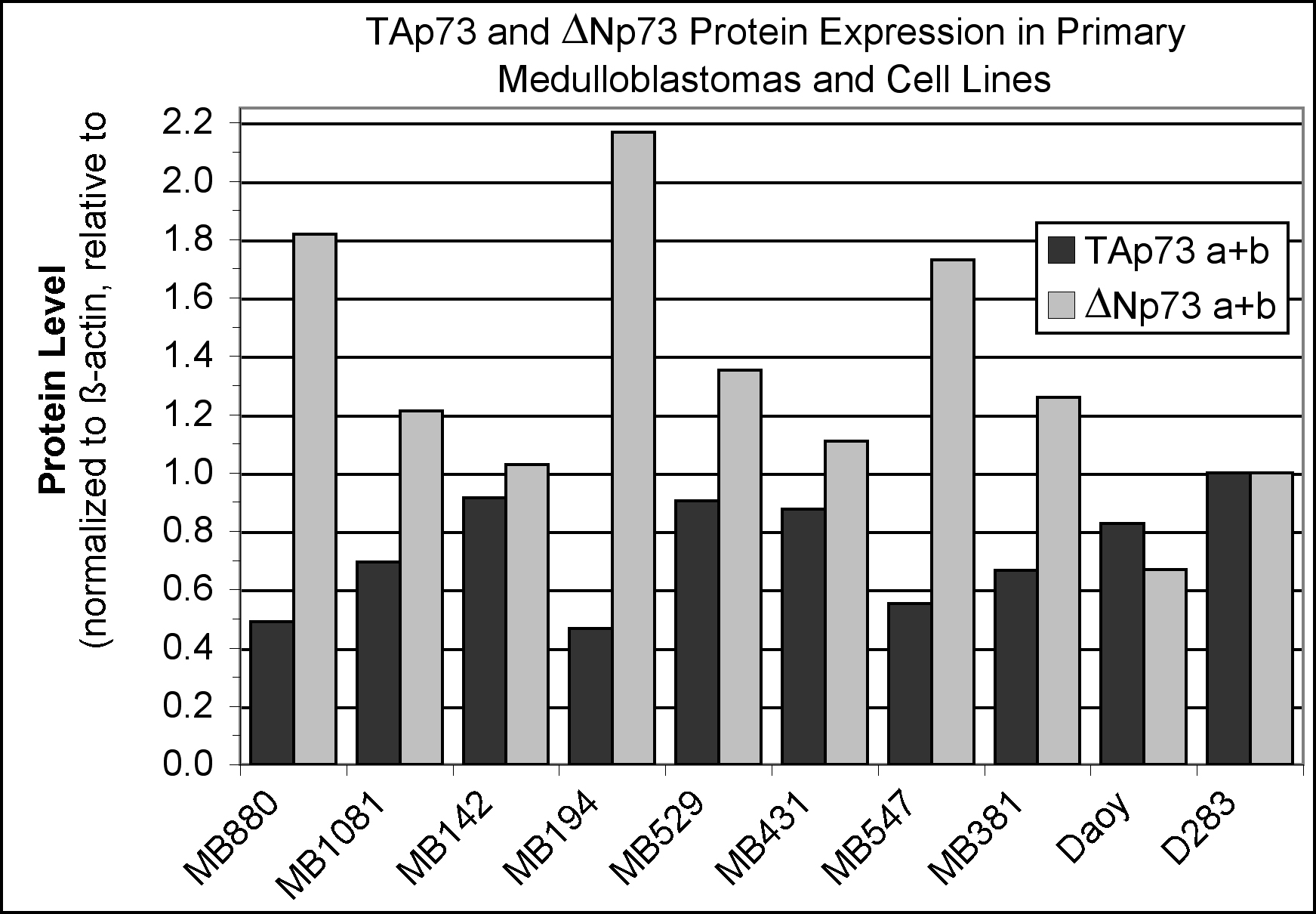

Supplement: Additional file 3 — TAp73 and ΔNp73 protein expression in primary medulloblastoma samples. Western blot analysis reveals that individual primary medulloblastoma samples from patients display overexpression of full-length TAp73 and amino-terminal truncated ΔNp73 (internally normalized to β-actin expression and relative to D283 cells). [file 1471-2407-7-127-S3.jpeg]

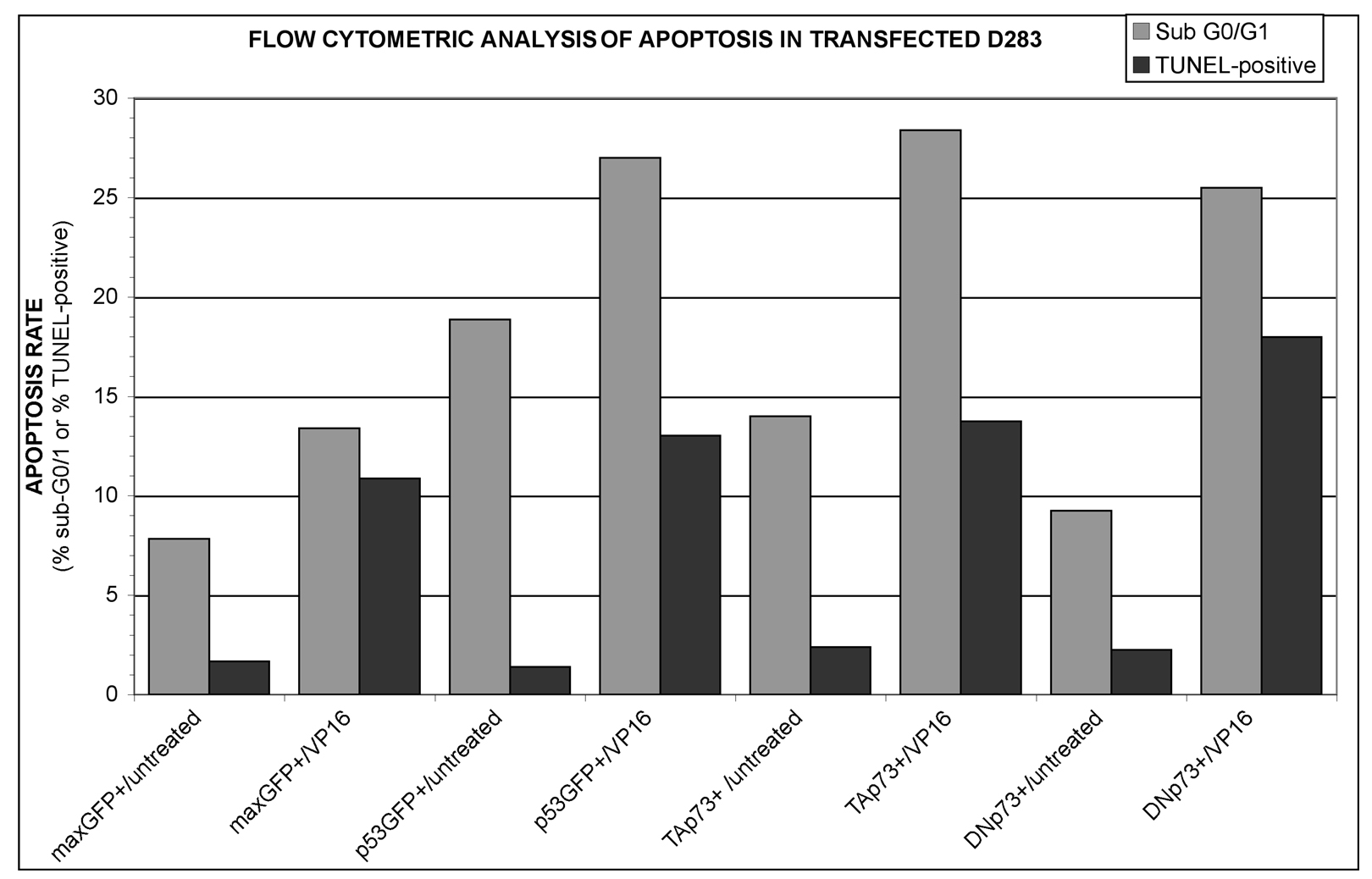

Supplement: Additional file 4 — Apoptosis analysis. TUNEL assay of D283 cell line confirms apoptotic changes in PI-stained cell populations in the sub-G0/G1 peak (i.e. hypodiploid (less than 2n) DNA content, representing apoptotic nuclei) detected using flow cytometric methods. X-axis, transfected plasmids (described in text) and post-transfection culture conditions (VP-16, 1.5 μM); Y-axis, % of cells with apoptotic features by flow cytometry or TUNEL assay. [file 1471-2407-7-127-S4.jpeg]
